# Picture naming test through the prism of cognitive neuroscience and linguistics: adapting the test for cerebellar tumor survivors—or pouring new wine in old sacks?

**DOI:** 10.3389/fpsyg.2024.1332391

**Published:** 2024-03-19

**Authors:** Olga Morkovina, Piruza Manukyan, Anastasia Sharapkova

**Affiliations:** ^1^Laboratory of Diagnostics and Advancing Cognitive Functions, Research Institute for Brain Development and Peak Performance, RUDN University, Moscow, Russia; ^2^Department of English, Faculty of Computational Mathematics and Cybernetics, Lomonosov Moscow State University, Moscow, Russia; ^3^Department of English Linguistics, Faculty of Philology, Lomonosov Moscow State University, Moscow, Russia

**Keywords:** picture naming test, neuropsychological assessment, lexical access, lexical retrieval, posterior fossa tumors, cerebellar tumor survivors

## Abstract

A picture naming test (PNT) has long been regarded as an integral part of neuropsychological assessment. In current research and clinical practice, it serves a variety of purposes. PNTs are used to assess the severity of speech impairment in aphasia, monitor possible cognitive decline in aging patients with or without age-related neurodegenerative disorders, track language development in children and map eloquent brain areas to be spared during surgery. In research settings, picture naming tests provide an insight into the process of lexical retrieval in monolingual and bilingual speakers. However, while numerous advances have occurred in linguistics and neuroscience since the classic, most widespread PNTs were developed, few of them have found their way into test design. Consequently, despite the popularity of PNTs in clinical and research practice, their relevance and objectivity remain questionable. The present study provides an overview of literature where relevant criticisms and concerns have been expressed over the recent decades. It aims to determine whether there is a significant gap between conventional test design and the current understanding of the mechanisms underlying lexical retrieval by focusing on the parameters that have been experimentally proven to influence picture naming. We discuss here the implications of these findings for improving and facilitating test design within the picture naming paradigm. Subsequently, we highlight the importance of designing specialized tests with a particular target group in mind, so that test variables could be selected for cerebellar tumor survivors.

## 1 Introduction

A picture naming test has long been a staple of neuropsychological assessment as it is relatively easy to perform and analyze (Laine and Martin, 2013). It has been shown to be sufficiently sensitive to language deficits of various etiology: stroke (Kertesz, 2007, 2020; Walker et al., 2022), traumatic brain injury (Barrow et al., 2006), tumors (Chakraborty et al., 2012; Papagno et al., 2023), or normal aging (Burke and Shafto, 2004; Strauss Hough, 2006; Macoir et al., 2017; Gallant et al., 2019). This has turned it into a useful research and clinical tool worldwide with various types, versions, and modes of running it.

However, its ubiquitous use in a variety of settings made it the most widely used tool to evaluate language deficits as well as the most questionable one in terms of interpreting and cross validating the obtained results, as they depend on 3 crucial variables: linguistic framework underlying the test, design principles and the underlying biological substrate of language deficits it might reveal. Meticulously balancing these variables is a tricky task to be confronted while devising any new test or adapting the earlier ones to specific groups.

Adherence to theoretically and empirically grounded principles in the development of PNT is particularly important when targeting under-represented groups without extensive research-based data to draw on. One group of particular interest is survivors of pediatric posterior fossa tumors (PFT), as central nervous system (CNS) tumors are the most prevalent of all solid tumors in children, and approximately 54–70% of all pediatric CNS tumors arise in the posterior fossa (Johnson et al., 2014). However, advances in modern medical practice have significantly increased the survival rates of children suffering from various oncological diseases, including CNS tumors. Studies investigating the impact of surviving cancer and its treatment have found that cognitive functions decline in pediatric PFT survivors (Hanzlik et al., 2015; Ahmadian et al., 2019). Thus, survivors are at risk for the long-term sequelae in neuropsychological functioning, which poses a risk to their long-term quality of life. The evidence for speech and language deficits in this group is still inconclusive, possibly due to the lack of the test accurately reflecting the patterns of deficits that result from PFTs and their treatments (Shurupova et al., 2021; Chipeeva et al., 2022). To effectively address this issue, it is essential to conduct an accurate assessment of language deficits in pediatric PFTs survivors, which may be overlooked in traditional tests. This is critical for the developing targeted interventions to address the specific language challenges faced by the children. To fill the gap of solid principles for devising the specific test that are lacking, we scrutinize its major components.

The major objective of this review is therefore to analyze the existing approaches to picture naming, outline their evolution over time, and suggest possible improvements to the method in the light of recent advances in cognitive neuroscience and linguistics. Not only are language deficits likely to involve various aspects even in case of a single locus of damage (which is indeed characteristic of most aphasias: e.g. Mikadze et al., 2018; Dronkers and Ivanova, 2023), but they are also likely to be accompanied by non-linguistic impairments.

Thus, a picture naming test, serving a simplified model of speech production being seemingly simple still represents a complex cognitive processes including a visual recognition of a stimulus, cognitive mapping of an item to a concept, lexical-semantic retrieval and finally articulation itself. Here, we conducted a comparative analysis of pertinent literature seeking for a more informed understanding of both neurocognitive and linguistic mechanisms underlying the picture naming test. The following databases were used for conducting the initial search for peer-reviewed articles: PubMed and Google Scholar (for the English language studies), and eLibrary (for the Russian language studies) using combinations of the relevant keywords, including “picture naming test”, “lexical access”, “lexical retrieval”, “posterior fossa tumor”, “cerebellar tumor”. Studies were selected based on their relevance to the topic and their contribution to understanding the theoretical frameworks of the Picture Naming Test in relation to models of speech and language processing. Additionally, we selected studies from the reference lists of the relevant articles to identify the earlier studies. After the initial search and identification of the key papers on the topic, we utilized Litmaps, Semantic Scholar, and Connected papers to identify and fill any gap in the literature search. Though the initial approach was comprehensive, we tried to limit ourselves to the most representative articles we cite in the present review.

We collated theoretical underpinnings from two lines of research—cognitive neuroscience and linguistics to settle the common ground for designing tests specifically targeting the understudied groups. Section 2 focuses on the history of the method itself and its variable applications and ways if running them. Test design is reviewed and key variables are listed within the context of their implications. Traditional tests and their more recent competitors in the field are considered in terms of possible inaccuracies or misinterpretations inherent in the design flaws stemming from. Section 3 zooms in on the theoretical foundations of PNT, which could be built on two lines of evidence. Section 4 provides our discussion based on the findings, followed by problematizing the new tests to be created. Finally, a number of linguistic and psychological improvements are also suggested, with the aim of providing the basis for a more specific, fine-tuned test. Most crucially, in this paper we try to propose principles for designing the test capable of identifying lexical disorders in children who survived posterior fossa tumors.

## 2 Historical and methodological insights into PNT

### 2.1 Historical insights into PNT and its applications

In the first half of the XX century, picture naming had already been viewed as a viable model of linguistic and cognitive functioning.

The earliest instances of its use date back to the 1880s, when a naming test was implemented to determine the type of item to be named most easily (Cattel, 1886). Next, attempts were made to use it as a diagnostic tool for measuring language impairment (Hawthorne, 1934), educational progress (Gates, 1924) or general intelligence (Binet and Simon, 1916; Terman and Chamberlain, 1918; Gregory, 2004). Conversely, some early studies utilized picture naming as a supplementary paradigm: for instance, to establish the preferred reading direction (Smith, 1932), foreign language aptitude (Carroll, 1958) or difference in preschoolers’ group behaviors (Harris, 1946). Action naming was also first applied in the early XX century as a means of revealing the link between children’s first-person experience (i.e., knowledge of objects and actions denoted by written words) and reading achievement (Woody, 1938). By the 1920s, both physical object and picture naming entered most batteries of language and cognitive assessment (Kuhlmann, 1922; Head, 1926), and the 1930s saw the appearance of the first standardized tests (Weisenburg and McBride, 1935). Since then, PNT has remained integral to language research and clinical practice.

Diagnostic PNTs are used to assess the severity and type of language impairment in people with aphasia or other deficits, including hearing loss (Jerger et al., 2002; Pellowski and Conture, 2005), stuttering (Maxfield et al., 2015), Alzheimer’s disease and dementia (Gallant et al., 2019). In a broader context, PNTs can be used as a measure of general intelligence (Differential Abilities Scale-II: Naming Vocabulary (Elliott et al., 2018); Clinical Evaluation of Language Fundamentals—Expressive Vocabulary (Wiig et al., 2013); or other cognitive skills (functions or domains), such as semantic memory in dementia or Alzheimer’s disease (the 64-item naming test from the Cambridge Semantic Battery)). Moreover, the XXI century saw the expansion of PNTs even to an operation suite. Peri- and intraoperative PN tests serve to map eloquent brain areas just prior to or during brain surgery (Mandonnet and Herbet, 2021).

Some general-purpose batteries, such as Folstein Mini-Mental State Examination (Folstein et al., 1975) or the Naming subtest of the Neuropsychological Assessment Battery (NAB) (Stern and White, 2004) can include brief picture naming tasks consisting of several items. In addition, cued picture naming can even be used for aphasia treatment (Wisenburn and Mahoney, 2009), and most clinical tests can be used in research and vice versa. The most prominent example is the Boston Naming Test (Riva et al., 2000; Bortnik et al., 2013), used both in clinical practice and research worldwide. Similar test paradigms can be found in other aphasia batteries: the Minnesota Test for Differential Diagnosis of Aphasia, Western Aphasia Battery-Revised (20 items), Philadelphia Naming Test (1996, 175 items), the Comprehensive Aphasia Test (see Fergadiotis et al., 2019) and the Northwestern Assessment of Verbs and Sentences (Cho-Reyes and Thompson, 2012).

Given this variety of purposes and settings, needs of specific populations cannot be neglected either. Tests such as Words in Game or Test of Word Finding are aimed at certain age groups, and special paradigms have been developed for bilinguals (see Bilingual Aphasia Test: Paradis, 2011; MINT: Gollan et al., 2012; or the ICMR-PNT: Paplikar et al., 2021, 2022). Nevertheless, adult tests are frequently administered to children—the BNT first and foremost. Standardization is typically, especially for peri- and intraoperative tests, which range from homemade batteries to non-specific assessments (Talacchi et al., 2013; Papatzalas et al., 2021). There are also recent attempts to devise a test for patients with Alzheimer’s diseases in native languages revealing that not only each language should be validated but also some specific ideas are to be taken into account specifically dealing within a clinical setting (Namdar Khatibani et al., 2020).

However, even the universally accepted PNTs remain controversial calling for a profound understanding of the processes contributing to picture naming since picture naming is orchestrated by a number of cognitive processes, ranging from visual recognition of a stimulus to lexical-semantic retrieval and finally to articulation. Meanwhile, most of these tests were developed prior to recent advances in neuroimaging, cognitive and corpus linguistics. Thus, if a picture naming test is to retain its role as a diagnostic method, several factors should be taken into account in devising the novel ones as well as interpreting the data from the classical ones: psycholinguistic theories of lexical access, corpus-based frequency data and the knowledge of underlying mechanisms of speech disorders or the possible neural correlates of picture naming.

### 2.2 Paradigms in PNT

The link between visual processing and language production has always been evident to researchers, resulting in two distinct paradigms. While both are used to assess lexical retrieval triggered by visual stimuli, they differ in purpose and procedure as well as the aspects of naming to be assessed. One paradigm requires rapid naming, whereas the other—which is the focus of the present review—does not focus on speed, recording the answers orally or asking the participants to write them. It goes without saying that interpreting the results of tests performed within different paradigms will be different.

In Rapid Picture Naming (or Rapid Automatized Naming), several images are represented on the page, and the participant is asked to name them as quickly as possible. While the cognitive mechanisms behind this connection are not fully explored (Decker et al., 2013), RAN is generally regarded as a predictor of reading performance—a correlation demonstrated already at the dawn of the naming method itself (Araújo et al., 2015). In contrast, confrontation naming does not emphasize speed, although time limits are still suggested for cueing and/or presenting the next item (Kaplan et al., 1983; Kertesz, 2007), and in surgery-associated testing procedures naming latency might indicates postoperative effects (Torrance et al., 2017; Mandonnet and Herbet, 2021; Miller et al., 2021).

As a rule, the answers are given orally and then recorded, but written responses might be elicited too (Torrance et al., 2017; Miller et al., 2021). For vision-impaired participants, the mode of presentation might be switched to auditory, involving different processes, and a sign language paradigm is used with patients with hearing loss (Navarrete et al., 2017; McGarry et al., 2020). The stimuli in confrontation naming are presented sequentially and do not repeat, and both object and action naming can be tested.

Relying on our current understanding of cognitive and linguistic functions, confrontation naming can perform two distinct functions. First, it is a reliable indicator of vocabulary size—and, therefore, of language development. This aspect is particularly valuable when assessing young children: even more so because naming sometimes serves as a predictor of literacy skills (see Missall et al., 2007). Popular tests of this type include the Expressive One Word Picture Vocabulary Test (190 items) and the Expressive Vocabulary Test (190 items).

Alternatively, naming problems might be pointing at a deficit at the level of language mechanisms proper. It is this very aspect that primarily attracts attention of linguists and neuroscientists alike. If naming is indeed a reliable indicator of deeper language deficits, then a simple test can reveal them, providing a broader picture for researchers and practitioners. However, to achieve this reliability, the link between naming and language mechanism must be explored and embodied in a clear model.

## 3 Conceptual underpinnings behind picture naming

### 3.1 Psycholinguistic models of lexical access

The assumption that a confrontation naming test can reflect a fundamental disruption in the language system functioning is rooted in certain views on lexical access in picture naming and its underlying cognitive mechanisms. Consequently, to be considered a relevant diagnostic method, a PNT must embody a distinct model of lexical access allowing for viable interpretation. However, the earliest tests did not appear to rely on a sound theory of speech production (and lexical access as its constituent part). In fact, it was not until the 1970s that the term itself was coined, first applied to visual word recognition—i.e. perception rather than production (Forster and Chambers, 1973). The models that have emerged since then, whether historical or currently relevant, can be classified according to different principles: “one-step” vs. “two-step”; cascading vs. discrete (Herbert, 2004); serial versus interactive; dual-stream vs. parallel models (Kerr et al., 2022); componential vs. holistic (Caramazza, 1997).

In the first half of the XX century, the link between language and mind was rarely formalized, despite being discussed in psychology and linguistics. Yet most of the early research stayed close to what was later termed a “mind in the mouth” approach (Bock, 1996)—a presumption of a direct one-to-one correspondence between the concept and speech output. Consequently, no clear-cut model of word retrieval seemed to have existed; mid-century works on picture naming, despite their meticulous procedure, made no reference to their theoretical underpinnings (e.g., Ervin, 1961; Oldfield and Wingfield, 1965).

The second half of the century saw the arrival of the first detailed theories of speech production. In (Fromkin, 1971), speech production was already regarded as stratified, and speech errors can be used to identify breakdown at a particular level. Next, Garrett’s model followed, suggesting a similarly structured top-down approach from semantics all the way down to articulation—with subsequent levels of processing accessed in turn, only once processing on the previous level has been finished (Garrett, 1975).

This serial structure remained prevalent in later research, most notably in the model developed by Levelt et al. (1998). It claims that activation during lexical access proceeds from the concept (semantic level) to lemma (lexical level) and to its morphological, phonological and, finally, articulatory shape. However, in more recent sequential models, known as cascading, overlap is allowed if activation starts simultaneously at adjacent lower levels (Morsella and Miozzo, 2002). Interactive models go even further, allowing for activation to spread from a lower to a higher adjacent level, progressing from phonology to semantics (Dell, 1986).

More recent models might refine or challenge the modular nature that is evident in both. The prime example is the hierarchical feedback control model, which combines the traditional hierarchy of levels with a more detailed understanding of neural mechanisms underlying it (Hickok, 2012). Another development, the parallel assembly model, presumes that different components of lexical access are processed simultaneously rather than step by step (hence “parallel”) and are then joined into a single whole—the “neural image”, or Gestalt, of a word as a single whole (Kerr et al., 2022).

Apart from postulated (non-)linearity of processing, another historically significant distinction is to be mentioned: namely, “one-step” versus “two-step” models. In the former group, competing for dominance until the 2000s, only two levels of processing used to be postulated: lexical-semantic and phonological (Stemberger, 1985; Starreveld and La Heij, 1995; Caramazza, 1997). In contrast, “two-level” models proposed an intermediate stage: the lemma level, where morphosyntactic features are stored, as opposed to the specific lexeme with its phonological and phonetic features to be eхpressed through articulatory or orthographic means (Dell and O’Seaghdha, 1992; Levelt et al., 1999). The term “lemma” itself originated in the late 1970s (Kempen and Hoenkamp, 1979, 1987); since then, “two-level” models are now accepted almost universally, the prime example being (Levelt et al., 1998).

While this debate can be seen as resolved, the organization of the lexical-semantic level is still far from consensus in modern cognitive linguistics (Pulman, 2005; Redmann et al., 2014). In componential, or decompositional models (Roelofs, 1992), the lexical meaning consists of distinct semantic nodes (Dell, 1986; Dell et al., 1997). Conversely, holistic (non-decompositional) models assume a one-to-one correspondence between lexical meaning and the underlying concept (Jescheniak and Levelt, 1994).

The variety of models listed above can be accounted for by the abundance and diversity of empirical data to build on, as well its source and type. Early models were mostly derived from aphasic data or speech errors of healthy speakers (Fromkin, 1971; Dell, 1986)—a method that has remained in wide use ever since (Gvion and Biran, 2023). Levelt’s model, in contrast, relied on healthy speakers’ picture naming latencies rather than accuracy of their responses (Levelt et al., 1999; Kerr et al., 2022). Another source is a “tip-of-the-tongue” state (ToT): a word retrieval difficulty in which “one cannot quite recall a familiar word but can recall words of similar form and meaning” (Brown and McNeill, 1966). It is observed in healthy as well as aphasic speakers, both in natural communication and controlled picture naming (Beeson et al., 1997). ToTs served as evidence of modular lexical access, specifically the lemma/word form distinction (Levelt, 1996; Levelt et al., 1999; Hofferberth-Sauer and Abrams, 2014; Abrams and Davis, 2016). Finally, the most obvious way of investigating neural mechanisms of lexical retrieval is by testing the brain directly during intraoperative testing by direct electric stimulation (DES). It can indeed differentiate between semantic, purely lexical and phonological levels (Mandonnet and Herbet, 2021). However, the need for more detailed tests controlled for a number of variables has already been voiced, as currently existing PNTs may be lacking accuracy (Rofes et al., 2015).

The diagnostic value of naming tests relies largely on the serial view of lexical access. Thus, the type of naming deficit, if observed in isolation, can be used to identify the locus of the deficit—in the conceptual, semantic or phonological level, or their interface (Friedmann et al., 2013). In clinical settings, this distinction frequently forms the basis of differential diagnosis and subsequent treatment of word-finding difficulties (German and Newman, 2004; German, 2015). Yet with the neuroimaging methods currently available, the next step has to involve establishing neural patterns behind each level—which, however, has not been achieved yet. The picture naming paradigm itself in its traditional form might not activate the same mechanisms as lexical retrieval in communication. Relying on purely visual objects while ignoring spatial or social clues as well as linguistic context appears to activate a different set of brain areas than observed in real-life interaction (Fekonja et al., 2021; Roos et al., 2023).

Recent advances in neuroimaging shed more light on the mechanisms of naming—in some cases, more detailed than purely clinical or linguistic data that was available earlier. However, most aspects require further clarification: for instance, both serial and cascaded models have been supported by empirical evidence (Schiller and Alario, 2023), as well as parallel processing (; Feng et al., 2021). Experimental data also points at some limitations of the paradigm, which does not fully reflect the mechanisms of lexical retrieval in connected speech, even if the perceived speech outcomes are comparable (Alyahya et al., 2021; Biran et al., 2023). Certain models of lexical access might not be present in some groups, such as children of preschool age, which have not been shown to possess the cascading mechanisms of lexical access observed in older speakers (Kandel and Snedeker, 2023).

Thus, research data on language processing and production show a considerable variability in modeling approaches and conceptual frameworks utilized. This has led to an ongoing debate regarding the preference of one particular model over the other, with no definitive data conclusively confirming the dominance of either. As a result, this lack of consistency presents a significant challenge when interpreting test results, since an experimenter’s choice of theory and model shapes the lens through which the data is analyzed and inferred.

With recent advances in neuroimaging, the links between language production elicited via picture naming tests and the underlying neural mechanisms become increasingly questionable. The parallel assembly model, for instance, is hardly consistent with some traditional assumptions:

- With a single word regarded as a distributed neural assembly—a “Gestalt” (Strijkers, 2016), the clearly stratified view of lexical access has to be discarded, at least partially—as lexical features in this model do not seem to exist *per se*, apart from sound and meaning (Kerr et al., 2022). This, in turn, would require a more careful reconsideration of error types according to the postulated level;
- The involvement of various brain areas in language processing suggest that the regions previously considered irrelevant might, in fact, play a significant part—and, therefore, damage to these areas might result in a language deficit even if the “primary” language areas are intact. Recent evidence suggests that instead of being stored in the brain as amodal sets of features, concepts remain modality-specific. Thus, processing words with perceptual-motor semantics—i.e. sensory, motor or emotional components—activates brain areas responsible for each (Kuhnke et al., 2023). This simultaneously supports the Lurian approach, which puts language deficits into a broader perspective, and the paradigm of grounded cognition (Barsalou, 2020). Within this approach, cognition is viewed as a module that, instead of being isolated, is intrinsically connected with physical perception and action, social interactions and the environment.^1^

While the mechanisms of lexical access still require detailed study, and no consensus on the preferred model has been reached yet, it is evident that current theories and developments in the field should be retrospectively applied to research paradigms developed prior to their arrival.

### 3.2 Lexical access revealed through instrumental methods

The changes in our understanding mentioned above became possible due to the advent of neuroimaging. Until recently, lexical retrieval models were mainly informed by linguistic data. Now, traditional models of lexical access can be verified and the new ones suggested on the basis of techniques such as ERP, MEG, fMRI, dMRI, PET or a combination of them. These techniques are often used to monitor brain activity during a picture naming test on-line unveiling much of what was previously unknown about the cognitive and neural architecture of speech processing.

One of the earliest techniques is ERP, employed since the 1980s (Stuss et al., 1986) to investigate brain function and localization during picture naming or identify activation times for different stages (Indefrey and Levelt, 2004). The authors identified brain regions that selectively respond to the naming task and provided insight into the temporal dynamics of lexical processing during naming performance (Indefrey and Levelt, 2004). It has proven useful in clarifying the structure of lexical retrieval. It first proved that grammar and phonology are encoded not only separately but also sequentially, so that phonology cannot be accessed apart from syntax (van Turennout et al., 1998)—while aphasic data could only show separate encoding of syntax. Despite its long history, ERP remains fit for the purpose: for example, by supporting parallel rather than strictly sequential activation (Feng et al., 2021).

To localize language functions, a number of other techniques are used, such as MEG (Salmelin et al., 1994), PET (Metter et al., 1981; Murtha et al., 1999; Catricalà et al., 2020) or fMRI (McCarthy et al., 1993; Hernandez et al., 2000), which show activation in specific brain areas evidenced by an increased blood flow. Another way would be to analyze the changes in speech produced by a “malfunctioning” area. In aphasia, this can be achieved through voxel-based lesion mapping (Saygın et al., 2004; Baldo et al., 2013; Dell et al., 2013; Snyder et al., 2023) and in healthy participants similar effect are imitated with TMS (Wassermann et al., 1999; Giampiccolo et al., 2020; Ward et al., 2022).

More than one technique might be combined in the study: take MRI and MEG, each efficient as a standalone method, which together provide more detailed spatiotemporal information (Alekseeva et al., 2022), or fMRI and dMRI used to map gray matter regions alongside fiber bundles (Jarret et al., 2022). Eventually, data gathered via neuroimaging could lead to the emergence of further models of lexical retrieval. This was the case with the “data-stream” model, which has become widespread over the last two decades: a combination of two networks in the brain—phonological (dorsal) and lexical-semantic processing (ventral)—instead of a single hierarchy (Dell et al., 2013). The ventral stream maps image and sound into meaning, while the dorsal stream links sound to articulation (Jarret et al., 2022). In its advanced form, sensory (auditory) and motor components differ at the phonological level, and production involves immediate feedback and control. Without an improved understanding of the brain, this model would hardly have emerged—similar to the parallel assembly model.

There are still some limitations to what non-invasive techniques can reveal. In some aspects, such as highlighting crucial language areas or dealing with individual differences, none can really compete against DES (Penfield and Rasmussen, 1950; Duffau et al., 2004). Nevertheless, due to the sheer range of methods now made available, Lakoff’s commitment to being “faithful to empirical discoveries about the nature of the mind/brain” (Lakoff, 1990) appears more demanding than ever—yet few of the picture naming tests currently in use were designed with a model of lexical retrieval in mind. An example of such conscious approach to design is the Test of Word Finding (German and Newman, 2004), where Levelt’s modified model serves as a theoretical basis for classifying test takers’ errors by the level they are processed on. Earlier paradigms, however, lack conscious theoretical foundations despite substantial clinical evidence. This poses challenges in interpreting the data for clinical and research purposes—especially if picture naming is regarded as a “window into the brain”, capable of indicating neurological deficits.

Speech disorders detected by confrontation naming tests can be correlated with specific brain lesions in a more accurate way—and, most importantly, they can be regarded as part of the global picture rather than isolated phenomena. This suggests that these deficits may be either symptoms resulting directly from brain lesions or secondary symptoms resulting from broader systemic reconfigurations. For example, cerebellar lesions lead to the breakdown of motor planning, which in turn affect speech. At the same time, similar symptoms do not necessarily indicate similar causes, and this requires particular attention to be paid to the disorder as a whole, not merely as a set of perceived abnormalities. Different lesions might be manifested in a similar set of perceived speech disturbances: for instance, cerebellar speech disorders have been reported to resemble transcortical motor aphasia, which results from a lesion in the frontal lobe of the dominant hemisphere (Mariën et al., 1996, 2000). While this should be taken into account when interpreting the results, the test, vice versa, must be designed with specific impairments in mind. A framework for designing such tests will be proposed later.

### 3.3 PNTs and current linguistic views on lexical retrieval

Recent advances in linguistics also have called for a more critical approach to PNT design and stimuli selection. Consequently, both validity and appropriacy of existing PNTs have repeatedly been questioned—first and foremost the BNT, which is still used most commonly worldwide as the seemingly universal magic pill to test various things at once.

The initial version of the test (1978) contained 85 stimuli, and in 1983 it was abridged to 60 items. This variant has remained common ever since. Some of the stimuli, however, have been regarded as culturally insensitive (noose: Lichtenstein et al., 2019; Eloi et al., 2021; Salo et al., 2021) and others showing significant effects of gender, age (especially in dated low-frequency items such as trellis or pretzel: Randolph et al., 1999; Schmitter-Edgecombe et al., 2000; Martielli and Blackburn, 2015), race (Azrin et al., 1996) and culture (Barker-Collo, 2001; Li et al., 2022). The original test lacks protection against culture and language bias: as a result, a variable might be crucial in one setting while insignificant in another.

Age in particular presents a problem in BNT-based studies. In one study of early aging effects on naming, participants aged 75 and older outperformed younger groups due to historical items such as yoke, trellis or abacus, which proved challenging to the rest (Schmitter-Edgecombe et al., 2000). No such effect, however, was observed within a senior group of Chinese participants tested on a 30-item adaptation, which included some of the problematic items (abacus, trellis: Chen et al., 2014). Younger participants tend to misinterpret some items in the adult version (Martielli and Blackburn, 2015), teenagers identified a funnel, rarely seen in a XXI-century household, as a martini glass. The BNT thus seems to measure lexicon size rather than naming ability and is additionally biased toward higher educational and intellectual level (Hawkins and Bender, 2002).

Other factors that are potentially significant for stimuli selection and arrangement must have been overlooked in the design of the BNT. The choice of semantic categories has not been explicitly controlled, which might obscure possible deficits in categorizing (Ardila, 2007; Harry and Crowe, 2014). While the stimuli are supposed to be ranged by increasing difficulty, the authors’ claim is not supported by evidence, as “difficulty”—whether based on everyday experience, visual complexity or lexical parameters—is never specified (Kaplan et al., 1983).

Psychometric properties of the BNT have also prompted criticism, being hardly consistent with the present-day standards. The lack of inter-rater reliability data (Harry and Crowe, 2014) as well as test–retest reliability analysis makes data collected using BNT unreliable. The issue is further complicated by the fact that the test is rarely administered according to recommendations; neither is possible ethnic, cultural and educational interference taken into account to adjust the score (Bortnik et al., 2013). With all that said, alternative tests created around the same time as the BNT are likely to share the same deficiencies, whereas later batteries such as NAB are not yet supported by sufficient data and might be hard to administer.

Any given PNT, by definition, consists of two levels: a set of visual stimuli and corresponding lexemes to be elicited. Both are likely to influence naming latency and accuracy, corresponding to different levels in lexical access where various parameters come into play. The most significant of them seem to be conceptual familiarity, visual complexity, imageability, image agreement, name agreement, age of acquisition, lexical frequency and word length. Visual complexity and image agreement determine adequate recognition of the object, while the semantic level is determined by imageability, concept familiarity and age of acquisition, which is also involved in phonological processing; name agreement applies to all three levels. Once motor programming starts, effects of word length (syllable / phonemic) set in (Heikkola et al., 2021). Several additional variables might influence lexical retrieval too: namely, phonological neighborhood density (Arutiunian and Lopukhina, 2018), type of object (natural or artificial), body-object interaction (BOI) and manipulability (Georgiou et al., 2022)—the latter two fitting the paradigm of grounded cognition.

The effect of most variables is confirmed by a number of studies. Nevertheless, some of them appear language-specific, including phonological neighborhood density and syllable length (see Bates et al., 2003; Arutiunian and Lopukhina, 2018; Perret and Bonin, 2018). Another important factor is age: while syllable length might affect naming in English-speaking children (James et al., 2016), it does not seem to do so in adults—unlike image and name agreement, imageability and age of acquisition (Perret and Bonin, 2018).

Selecting stimuli for a PNT from a standardized database would reveal the role of each variable under controlled conditions. The first psycholinguistic databases for imageability (Walker, 1970), concept familiarity (Bowen, 1969; Stration et al., 1975) and age of acquisition (Carroll and White, 1973b) appeared in the middle of the XX century, and soon attempts at creating psycholinguistically sound stimuli sets were made—the most prominent being the Snodgrass-Vanderwart database (Snodgrass and Vanderwart, 1980). Unlike earlier sets, it was standardized by familiarity, image agreement, name agreement (for English) and visual complexity. Later the database was adapted for other languages and cultures (Szekely et al., 2004), including Russian (Tsaparina et al., 2011). New objects were added and, for some adaptations, colored pictures used instead of the original black and white line drawings to increase ecological validity (Rossion and Pourtois, 2004).

Extensive databases of colored pictures and photographs such as BOSS (Brodeur et al., 2010) arrived next, followed by alternative sets (Viggiano et al., 2004; Adlington et al., 2009; Moreno-Martínez et al., 2011; Duñabeitia et al., 2018). Both seem to improve accuracy and latency of naming—presumably due to additional visual cues such as color, texture and shading compared to black and white line drawings. The difference between colored pictures and photographs, however, has not been universally evidenced (see Reymond et al., 2022). Neither, however, has affected the current state of affairs in the testing field, since most of the widely used PNTs (the BNT among them) feature their own picture sets, making inter-test comparison impossible (Bonin et al., 2003). With the arrival of virtual and augmented reality, 3D sets are becoming feasible (Peeters, 2018), potentially bringing back the practice of using tangible real-life objects as stimuli (e.g., Head, 1926).

Among the variables associated with language units, lexical frequency remains much disputed. Since its first mention as a significant psycholinguistic variable in the 1950s (Howes and Solomon, 1951), it has been included in numerous studies—from some of the earliest works (e.g., Oldfield and Wingfield, 1965) to the recent ones, including research into neural activation (Strijkers et al., 2017; Fairs et al., 2021). However, few of the existing tests employ frequency norms consistently, presumably due to the absence of spoken frequency estimates at the time of their development. Earlier studies used to rely on Kučera & Francis’ list, drawn from the 1-million corpus of written English texts published in 1961—the Brown corpus (Kučera and Francis, 1967). Considering the nature of the texts in the Brown Corpus, as well as their timespan, they hardly reflected the actual frequency of target items in everyday speech (to say nothing of children’s output). Yet even these norms were not reflected in the design of some popular PNTs, notably the BNT (Yochim et al., 2013) and the NAB, which, with its emphasis on spoken language, was apparently meant to ignore them altogether (Roach et al., 1996). The Philadelphia Naming Test (Roach et al., 1996) and various modifications of the Test of Word Finding (German, 1991, 2015) seem the exception to the rule (Morkovina et al., 2023).

Eventually, speech-based frequency estimates arrived with the first corpora: the late XX-century CELEX database of transcribed spoken interactions (Rofes et al., 2015), followed by HAL—a corpus of online communication with around 131 million entries (Burgess and Livesay, 1998). In 2009, the first database of fully spoken language—SUBTLEX (part of the Elexicon project)—was released, including film and television subtitles (Brysbaert and New, 2009). This was the start of a SUBTLEX family of corpora for Chinese (SUBTLEX-CH), Dutch (SUBTLEX-NL) and variants of English (SUBTLEX-US, SUBTLEX-UK). Subtitles have remained a convenient source of frequency data ever since because of sheer volume and representativeness, especially in the low-frequency domain (Fruehwald, 2017; Baranowski and Turton, 2020). Another approach would be to rely on social network for real-life interactions (Wordlex: Gimenes and New, 2015), including less conventional sources such as picture annotations (Petilli et al., 2022). Finally, the arrival of corpus managers allows comparison across corpora, which might prove particularly useful when dealing with bilingual data. With all these sources available, traditional PNTs prove particularly inconsistent with real-life frequency data—for instance, retrieved from the SUBTLEX-US Corpus (Yochim et al., 2013).

An equally (if not more) significant variable is age of acquisition (AoA), indeed supposed to underlie frequency effects and, therefore, be a more reliable predictor of naming efficiency (Carroll and White, 1973a,b; Morrison et al., 1992). Since the 1960s, when it was first singled out as a significant psycholinguistic variable (Rochford and Williams, 1962), two AoA measures have been introduced: subjective and objective. Subjective AoA is measured on the basis of adult speakers’ estimates using a Likert scale (Carroll and White, 1973a,b). Conversely, objective AoA directly addresses young speakers: the 75% rule might be used—namely, the age at which 75% of children can correctly retrieve the target item in a naming task (Morrison et al., 1992; Grigoriev and Oshhepkov, 2013). Although the effects of objective AoA are more pronounced, both subjective and objective age of acquisition can be used interchangeably (Elsherif et al., 2023).

Another variable to be accounted for is imageability (the ability to imagine an object denoted by the lexical unit in question). It is closely linked to concreteness—which is responsible for the so-called “concreteness effect”, which makes concrete nouns more easily memorable than abstract ones (Stoke, 1929; Gorman, 1961; Paivio, 1963; Paivio et al., 1968). Low-frequency words, such as “armadillo”, which form the basis of most naming tests, are usually characterized by high concreteness and low imageability (Kiran et al., 2009). Since mid-XX century, the concreteness norms have been established for a number of languages, including English (Brysbaert et al., 2014b), French (Bonin et al., 2018), Dutch (Brysbaert et al., 2014a), Chinese (Yao et al., 2016), Portuguese (Janczura et al., 2007; Soares et al., 2016) and some other languages. Yet it remains relevant in the paradigm of grounded cognition, being rooted in bodily experience.

The emergence of discourse studies coupled with cognitive linguistics may also be regarded as another factor to be considered in PNT development. Increased attention to spoken discourse as a system in its own right, with rules different from those of written language, might call for a more detailed attention to naming under test conditions contrasted with naming in actual discourse—which influences functional communication most, with isolated naming being viewed primarily as its indicator. Discourse informativeness, indeed, seems to correlate strongly with confrontation naming (such as communicating the gist of a narrative)—yet only in certain types of aphasia (Richardson et al., 2018; Tilton-Bolowsky et al., 2023; Li and Kiran, 2024). Beyond that, predictive powers of PNTs appear limited and are to be interpreted only in tandem with other methods prompting some coherent narrative production. For instance, when word-finding difficulties such as paraphasias are considered in isolation, correlation is negligible (Fergadiotis and Wright, 2015). Unlike picture naming, in discourse generation the target item is embedded in a complete utterance, which, despite the absence of visual stimuli to be processed, involves not only a broader range of cognitive functions—memory and executive functions, but also the impact of the linguistic context *per se*—grammar, morphosyntax, and collocability (Kim et al., 2019). Consequently, confrontation naming fails to capture conditions that are primarily related to cognitive status, such as MCI, in contrast to narrative/discourse samples. Given the importance of preclinical diagnostics in senior populations, the suitability of confrontation naming in this field was questioned in (Fleming and Harris, 2008). In the senior cohorts a combination of word retrieval measures, such as a PNT and a (semi-) spontaneous speech sample or fluency task, could be considered more viable (Martínez-Ferreiro, 2024).

Finally, the examiner’s proficiency in the language of testing should not be dismissted: otherwise, assessment errors and, in clinical populations, misdiagnosis might occur (Echemendia, 2005). Even relatively minor differences, such as regionalisms or slang in the subject’s dialect, could result in significant misinterpretation. Behavior during the test might be culture-bound as well: for instance, in Hispanics speed of response is rarely a priority (Ardila et al., 2002). Therefore, testing should be carried out preferably by native speakers, including bilingual assessments (Gollan et al., 2007, 2010), where meticulous protocols can be developed to ensure proficiency (Paplikar et al., 2021, 2022). In settings where neuropsychological testing is not universally applied, or in languages that have rarely been studied, this requirement might remain problematic (Hatahet et al., 2023).

All this is bound to influence both test design and interpretation of the results, as well as normative data. The need for more stringent tests, consistent with current developments in neuroscience and linguistics, is evident irrespective of the form of administration or test setting. Even intraoperative assessment, known to yield fairly reliable results, requires improved tests controlled for more variables.

**Figure 1:**
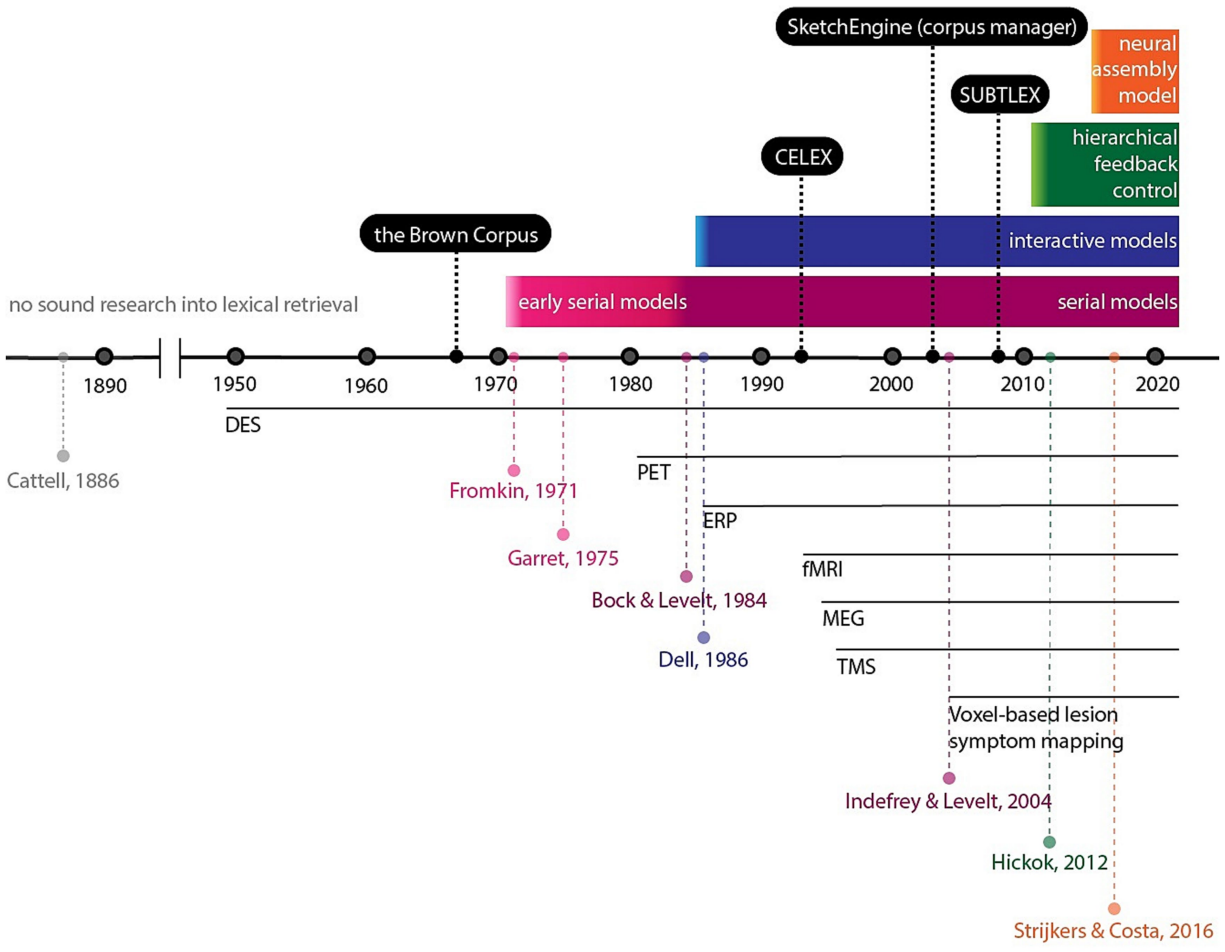
Picture naming test evolution and application at the background of theoretical models. Solid lines show the development of lexical access models explored in a number of articles. Below the timeline, horizontal lines indicate development of instrumental methods, starting from the earliest mention of a particular method used in tandem with a picture naming test. Above the timeline, corpus tools associated with picture naming are listed.

For heuristic purposes we tried to summarize the intricate and intertwining complexity behind the quest for a profound understanding of how lexical access within the context of linguistic and cognitive neuroscience in Figure 1.

## 4 Looking for some new wine to fill the old sacks: adaptations of and novelties in PNT

With all the above-mentioned issues in mind, improving PNTs in various aspects has been attempted. Overall, two major trends can be singled out: adapting the existing tests to new languages, cultures or participant groups, and, alternatively, designing the new ones.

The adaptation trend unsurprisingly started with the BNT. Versions of the BNT currently exist for different languages, including Swedish (Tallberg, 2005), Portuguese (Miotto et al., 2010), Chinese (Cheung et al., 2004) etc. While the early adaptations relied on a straightforward item-to-item translation (e.g., Mariën et al., 1998), in more recent versions culture-specific items such as plant and animal names, tools, musical instruments or folklore characters might be changed between languages (“unicorn” to “wayang”—“a shadow puppet”, “harmonica” to “seruling”—a musical instrument in the Indonesian version of the test; Sulastri et al., 2018) and even local varieties of the same language (“beaver” to “platypus” in Australian English: Cruice et al., 2000). Appropriate restructuring might be made when adapting the existing tests to new languages (Allegri et al., 1997; Kohnert et al., 1998; Vivas et al., 2020). In this case, at least some of the properties of specific items should be taken into account, such as lexical frequency, as they differ across languages. Alternatively, one could select a different test to suit a new task or setting. Nevertheless, most of the late XX-century picture naming tests such as the Graded Naming Test, NAB and the Philadelphia Naming Test fail to address all the issues of the BNT for historical reasons (Harry and Crowe, 2014).

With all this in mind, designing new PNTs seems to be more reasonable—which is proven by the growing number of newly developed tests. Some are created on a case-by-case basis for a specific research purpose (Gertel et al., 2020), while others are meant to replace the existing paradigms (French in Quebec: TDQ-60, TDQ-30—Macoir et al., 2017, 2021; Italian—Papagno et al., 2020; Dutch: DNT-92—Alons et al., 2022; German: CoNaT (although essentially a bank of items rather than a fully-fledged test)—Weiss Lucas et al., 2021) (Macoir et al., 2017, 2021; Papagno et al., 2020; Weiss Lucas et al., 2021; Alons et al., 2022). In most cases, a turn from the universal to the specific has taken place, so that the picture naming paradigm is adjusted to the language, culture, target group or clinical issue in question. This is particularly true for tests targeting specific groups, such as bilinguals, children or clinical populations. Thus, it is not the approach of “one size fits all” with one good test but rather a broad, more inclusive and targeted approach.

Bilingualism in particular appears to influence picture naming outcomes, sometimes more strongly than other paradigms (Gollan et al., 2005). Generally, it results in longer naming latencies and a lower accuracy compared to monolinguals (Gollan et al., 2005), while improving executive functioning and, possibly, slowing down the age-related cognitive decline (Craik et al., 2010). Even in clinical populations, language assessment in bilinguals can yield unpredictable results (Gollan et al., 2010). Cross-language effects are frequently observed, whether qualitative or quantitative (Gollan et al., 2002, 2007, 2012; Sheppard et al., 2016), and language-specific features might significantly influence the overall performance (Dai et al., 2012; Gollan et al., 2012). While the effects of bilingualism in picture naming offer valuable insight into the nature of language processing in general (Gollan et al., 2005; Strijkers, 2016), their clinical implications matter most, as naming is commonly used for diagnosing Alzheimer’s disease, dementia or MCI. Lower and middle income countries, where the incidence of cognitive disorders is expected to increase, are particularly at risk (Paplikar et al., 2021). Non-European settings are particularly vulnerable, given that most PNTs were originally developed for Western languages and cultures, non-European settings require special attention. This also applies to neuropsychological norms, of which PNTs are presumably indicative.

Consequently, conventional monolingual PNTs might elicit deficit-like patterns resulting in misdiagnosis, especially false-positive errors (Gollan et al., 2002, 2007), if applied without adequate adjustment, embracing linguistic and cultural peculiarities. The need for more clinically relevant testing paradigms arises, which would take into consideration the following:

1. Language dominance, balance and proficiency, which have been reported to influence performance on PNTs. Various measures of establishing the dominant language have been suggested: from self-rated questionnaires to proficiency interviews to further picture naming tests (Gollan et al., 2007, 2012; Tomoschuk et al., 2018; Zhou and Privitera, 2024). The value of each in isolation remaining questionable, combining could prove beneficial (Paplikar et al., 2021).
2. Context of language use should be accounted for, as even balanced bilinguals differ in their knowledge of items encountered in language of education or language of the home (Bialystok and Craik, 2007; Sheppard et al., 2016). This might result in varying item difficulty across languages (Bialystok and Craik, 2007; Gollan et al., 2007). This factor is particularly relevant in populations where multiple languages are used simultaneously (Paplikar et al., 2021).
3. Sociocultural conditions can affect item difficulty as well—for instance, the use of the abacus as an educational tool in China in contrast to English-speaking countries (Tomoschuk et al., 2018; Li et al., 2022). Nevertheless, this factor should not be overestimated, as it hardly accounts for the difference between bilingual and monolingual performance, even if controlled for (Misdraji-Hammond et al., 2014).
4. Testing methodology (including instructions, scoring and assessment guidelines) should be carefully selected to fit the purpose. A number of options are available: namely, the dominant or non-dominant language score, followed by either-language (i.e., with the answer given in one language accepted as correct) and both-language ones (Gollan et al., 2022). In mixed-language settings, further distinctions might apply, with borrowed words regarded as part of the target language (Paplikar et al., 2021) or two languages used interchangeably for sociocultural reasons (Ardila et al., 2002). Strict and lenient scoring of answers is also worth mentioning (Sheppard et al., 2016).
5. Target languages. The presence of cognates, i.e., phonologically related translation equivalents, has been reported to influence testing outcomes (Gollan et al., 2007), as well as basic structure of the languages spoken by participants (Dai et al., 2012). Both can result, alternatively, in interference or facilitation. Item difficulty in monolingual tests, such as the BNT, matches only the original language, which might lead to unexpected outcomes—such as better performance in English for non-English-dominant speakers (Gollan et al., 2012).

Cross-linguistic paradigms have been developed to remedy the issue. One of them is the Multilingual Naming Test (MINT), designed for bilingual speakers of English, Spanish, Mandarin Chinese and Hebrew, which has substituted the BNT in Alzheimer’s disease research since 2015 (Gollan et al., 2012; Stasenko et al., 2019). For mixed-language settings, a valid example is the ICMR-NCTB (Paplikar et al., 2021, 2022), developed for a range of languages spoken across India with cultural and social characteristics of the population in mind—which is fully reflected in the assessment and scoring protocol.

Testing children with conventional adult batteries is equally or even more unreliable. As a rule, tests for younger participants contain fewer items and are supposed to take less time to accommodate for a lower attention span and inability to stay on task for a prolonged time. This, however, does not eliminate the possibility of misinterpretation (see Martielli and Blackburn, 2015), likely to stem from the difference between adult and child language input. Corpora of child or child-directed speech could be used in designing naming tests that would reflect it; and attempts to single out a separate group of child-directed texts have been made since the middle of the century. The previously mentioned Thorndike & Lorge’s frequency list (Thorndike, 1944) included a selection of books for children and young adults (120 books, about 4.5 million occurrences) as a source. This principle of frequency list compilation would be maintained later (Zeno et al., 1995; Lété et al., 2004; Terzopoulos et al., 2017), to culminate in the creation of child-specific databases such as CHILDES (Child Language Data Exchange System; MacWhinney, 2014). However, none of the PNTs currently in use seem to have relied on this data. With clinical populations, the situation appears similar.

Another point to be considered is the role a particular scientific framework might play in the setting that the PNT in question is intended for. In Russian neuropsychological testing, for instance, similar trends toward universalism and specialization can be observed. However, the basic premises have differed from the start, with A.R. Luria’s syndrome approach dominating the field. His works were preceded by a clinical tradition which included detailed observations of speech abnormalities. These, including naming deficits, were viewed as complex phenomena involving different levels of processing (to the extent it could be understood at the time: see Bernshtein, 1912). When possible, speech abnormalities in different conditions were compared. The first steps toward a standardized examination protocol were made in the early XX century (Krol, 1933), followed by the publication and subsequent adoption of Henry Head’s tests in diagnostic practice (Head, 1926). The Lurian approach, first formed in the 1940s (Luria, 1947), differs fundamentally in its premises from the Western view of language processing. Instead of trying to map separate language levels or functions on clearly defined brain areas, Luria’s approach regards speech disorders as primary or secondary deficits in higher mental functions (Mikadze et al., 2018). Luria created a comprehensive system for the study of human cognitive functions and their brain organization. Diagnostics and rehabilitation procedures naturally follow from the theoretical bases of Lurian neuropsychology. There are two fundamental diagnostic principles in this tradition: (1) dysfunctions of different brain regions lead to diverse manifestations of impairment within a specific cognitive function; (2) dysfunction of a particular brain area leads to impairments not only in one but multiple functions, all following a unified principle. Speech, considered as one of the higher mental functions, should not be examined in isolation, instead it should be assessed within a comprehensive examination procedure that shows what functions are impaired and what functions are intact. Consequently, a different classification of speech and language disorders arises, where some of the syndromes have no direct equivalents in the Boston system.

The evidence behind Luria’s approach stemmed from younger patients with traumatic aphasia rather than senior patients with vascular lesions. Nevertheless, attempts have been made to combine different traditions, thus preserving the most relevant features of each. For instance, the Luria–Nebraska Neuropsychological Battery (Golden et al., 1980, 1985; Golden, 1987) is claimed to be a combination of Luria’s approach with some of the tenets of Western neuropsychology. However, in most cases the development of PNTs is rooted in a specific system, whether local or adopted. While the Lurian approach has been successfully adopted in Eastern Europe and Latin America, where it still prevails, some recent editions of Western batteries were adapted for Russian at the turn of the century [BDAE-3 (Goodglass et al., 2001), WAB-R (Kertesz, 2007), CAT (Swinburn et al., 2004), Aphasia Rapid Test (Buivolova et al., 2020)]. The most orthodox realization of Luria’s approach is MORA-1981, the most widespread Russian test battery (Tsvetkova et al., 1981). It gave rise to a number of subsequent tests, which transfer its principles to different populations or settings (e.g., Markovskaya, 1993; Vizel, 2005). They remain part of clinical practice (e.g., Semenova and Neznanov, 2022).

However, the previously mentioned deficiencies are also common to Russian tests. Until recently, little experimental data has been available to justify test design and stimuli selection, and psychometric properties of the existing paradigms are doubtful. Moreover, test materials are sometimes hard to obtain, and the quality of the pictures might suffer. In some cases, authors’ claims concerning frequency or familiarity with the target items are barely supported by evidence (Fotekova and Akhutina, 2002; Glozman et al., 2008). Certain variables, such as age of acquisition, were not available at the time the tests were created, and the absence of data still remains an obstacle. XX-century tests within the Lurian paradigm tend to rely on their own picture sets. Eventually, subjective AoA norms for colorized Snodgrass-Vanderwart pictures were established, yet not made public (Tsaparina et al., 2011), and a new normed stimuli set was released (Akinina et al., 2014). Objective AoA was partially explored by Grigoriev (Grigoriev and Oshhepkov, 2013). Frequency, in contrast, has been easy to estimate since 2008, when the Frequency dictionary was released (Lyashevskaya and Sharov, 2009). More information, covering collocations as well as single units, can be retrieved from the subcorpora of the Russian National corpus together with contexts of use. These and some other parameters, such as phonological neighborhood density, can be conveniently accessed via the StimulStat database (Alexeeva et al., 2018). Additionally, for 506 common nouns parameters linked with grounded cognition, such as manipulability, can be retrieved (Miklashevsky, 2017).

The current state of psycholinguistics, therefore, finally seems to allow for more reliable, psycholinguistically sound tests to be created. Some attempts have already been made: the Russian Aphasia Test (RAT: Ivanova et al., 2021) and the standardized Russian Child Language Assessment Battery (RuCLAB: Gomozova et al., 2021), the Russian Intraoperative Naming Test (Dragoy et al., 2016) etc. Unlike earlier batteries, these PNTs aim at contemporary psychometric standards and rely on relevant linguistic data. However, most of them are intended for a wide range of contexts rather than specific issues, which might potentially lower their sensitivity in cases that lie beyond the usual scope of aphasiology—for instance, deficits caused by pediatric cerebellar tumors.

## 5 Discussion

### 5.1 Adapting the test for clinical samples

One population of particular social importance is pediatric brain tumor survivors. Tumors of the central nervous system account for approximately 25% of childhood cancers, second only to leukemia (Cacciotti et al., 2020). Among these, tumors of the posterior cranial fossa rank first, where the cerebellum is most commonly affected. Since the survival rate of these patients has increased significantly with the development of multimodal therapies over the last 20 years (Suk et al., 2022), quality of life is now becoming a matter of concern. And with it, the problem of potential speech disorders that might involve nominative function. Yet speech and language disorders in children with tumors of the posterior cranial fossa are practically unstudied, which is probably due to both the peculiarities of diagnosing children and the long-held ideas disregarding the role of cerebellum in speech and language processing and generation confined to the purely motor level only with a fronto-temporal brain network implicated in language production and comprehension. Sample size is also a problem, since most studies cover small groups with less than 30 cases (e.g., Berger et al., 2005; Rønning et al., 2005; Vaquero et al., 2008).

Most studies concerning the cerebellum have focused on various disorders related to movement control. The impairment of the motor component of speech in cerebellar lesions was also noted as early as the first half of the twentieth century (Krol, 1933). However, recent data of neuroanatomical, neuroimaging and neuropsychological studies suggest that the cerebellum plays a leading role not only in motor functions, but also contributes to higher cognitive functions (Bellebaum and Daum, 2007). Moreover, these effects are likely to originate from the malfunctioning of a network of complex connections of cerebro-cerebellar and cerebello-cerebral connections (Schmahmann, 1991). The damage to these links results in a range of deficits beyond the motor level: adverse effects on memory, attention and executive functions have been reported, resulting in symptoms similar to those in autism, ADHD or developmental dyslexia (Stoodley and Limperopoulos, 2016). Cerebellar alterations of various etiology appear to influence cognitive functioning in a similar way as they influence motor control, resulting in “dysmetria of thought,” or the cerebellar cognitive affective syndrome (Schmahmann, 1996). While CCAS and the cerebellar motor syndrome are caused by lesions to different regions of the cerebellum, an almost identical inability to meet the intended target—whether spatial or cognitive—is observed in both (Schmahmann, 2022).

Along with other higher functions, language is frequently impaired in cerebellar cases. The most commonly known consequence of cerebellar damage is mutism, and less grave deficits include decreased verbal fluency, agrammatism, abnormal syntactic and prosodic structure, mild anomia and possible problems with metaphor and inference (Mariën et al., 2013; Schmahmann, 2016). Meanwhile, there is no consensus on whether these issues qualify as aphasia (see Mariën et al., 2013). A few studies harnessing various forms of PNTs to clinical samples have reported inconsistent results. Specifically, impairments in semantic retrieval have been observed in cerebellar stroke patients, with conversational skills mostly preserved (Fiez et al., 1992). Impairments in phonological but not semantic fluency have been observed in patients with focal or degenerative lesions of the left and right cerebellar hemispheres (Leggio et al., 2000). In patients with left or right cerebellar lesions, reduced fluency has been reported (Cook et al., 2004; Whelan and Murdoch, 2005), as well as dyslexia-like impairments (Moretti et al., 2002), and difficulty generating definitions for polysemous words (Murdoch and Whelan, 2007).

Since lexical access involves language processing at all levels, a PNT could be a valid method of evaluating the extent of speech and language deficits in cerebellar patients. Neuropsychological studies have already established functional correlations between specific areas of the cerebellum and the resulting motor, affective or cognitive damage (Mariën et al., 2013; Schmahmann, 2016). Still, despite the long history of its use to diagnose speech disorders in stroke, brain injury, and neurodegenerative diseases, it has not yet established its role in the diagnostic repertoire of neuro-oncology.

The evidence from traditional tests when applied to clinical samples is controversial. Some studies involving non-specific tests such as the Boston Test, Naming Vocabulary (DAS-II) subtest or EOWPVT-R vocabulary test (Levisohn et al., 2000) indicate speech disorders in isolated cerebellar lesions, including object naming, which correlates with neuroimaging data. In contrast, others do not find serious language deficits in children with cerebellar lesions (Hudson and Murdoch, 1992; Richter et al., 2005). However, data from cerebellar cases of different etiology might suggest some directions for further investigation. For instance, naming deficits were detected by means of a PNT in Friedreich’s ataxia: an autosomal recessive genetic disorder characterized by progressive atrophy of the cerebellar gray matter. Similarly to survivors of cerebellar tumors, the patients with this disorder experience ataxia when walking, impaired handwriting, dysarthria, and leg weakness. By administering a specially developed test, consisting of a set of nouns and verbs specifically selected from the International Picture Naming Project, the authors found no significant difference between the control group and the subject group in object naming, whereas action naming was clearly impaired (Nieto et al., 2012). Studies that do not explicitly use PNTs might prove useful as a starting point for further research, given the scarcity of studies of children samples. Comprehension, for instance, is normally intact, yet the correlation between IQ and comprehension of instructions in children with congenital cerebellar disorders suggests similar effects in the PFT population (Butti et al., 2023). Thus, using PNTs in neuro-oncology might prove fruitful—provided the tests are designed to suit both the age of the clinical population and the specific type of disorder: in that case, cerebellar tumors. When developing them, history and the current state of PNTs in the target language should be considered.

### 5.2 Developing a Russian test to target PFTs children

Given this theoretical and empirical backdrop, it seems reasonable to expect that a text designed for a population of PFT survivors is to be created on the well-grounded basis. To explore the extent of language impairment, a specialized test was designed to determine the role of sensorimotor components in lexical access. While its role in language processing was previously mentioned in academic literature (Migun and Spiridonov, 2018; Reifegerste et al., 2021), the effects for Russian have not been studied. We put forward the following propositions to be tested with the help of this paradigm.

Lexical access is likely to be carried out differently in:

- objects with / without sensorimotor semantics;
- static / dynamic body parts, involved in fine and gross motor skills;
- human / animal body parts;
- actions and objects (not) related to the speaker’s immediate sensorimotor experience;
- states / actions (with or without spatial limits);
- actions performed by human / non-human agents;
- actions with one or more agents of different types;
- emotionally charged actions.

The stimuli were retrieved from the “Noun and Object” and “Verb and Action” databases (Akinina et al., 2014), and additional images of non-existing objects were created specifically for the test. Each experimental series, representing objects and actions respectively, exists in 2 variants, made equivalent according to the key parameters, which is expected to increase test reliability. In either variant, paired stimuli are equated for the percentage of name agreement and the H index, imageability, lexical frequency, subjective age of acquisition, syllable and phoneme length. Several sensorimotor aspects have been selected for control in accordance with the hypotheses listed above: the speaker’s presumed experience of performing an action (for verbs) or interacting with the object (for nouns); the static or dynamic nature of the event denoted by the verb or associated with the object; the directionality of the movement and the involvement of gross and fine motor skills. The test covers a range of verbs (action / state; transitive / non-transitive; instrumental; reflexive) and nouns—particularly objective and instrumental. Another way of revealing the role of sensorimotor experience is comparing participants’ performance on identical items with referents different in sensorimotor experience, agents and objects (i.e., to scratch—when performed by a cat or by a human using an instrument).

Unlike traditional aphasiological batteries, the proposed test does not aim at diagnosing various speech disorders and differentiating between them. Its main purpose is to investigate lexical processing from the perspective of grounded cognition in healthy speakers as opposed to a carefully selected clinical group: survivors of pediatric posterior fossa tumors. It might, however, help to clarify additional aspects of lexical access and the influence of secondary variables, such as word length, age of acquisition, the presence of objective and instrumental semantics. Moreover, the test includes optional questions to investigate the grammatical component, whereas narrative and descriptive strategies could be revealed by participants’ responses to non-existent objects (the additional pictures)—both in isolation and compared against discourse generation tasks, such as summarizing a video (i.e., W. Chafe’s The Pear Film: Chafe, 1980).

An automated procedure based on dedicated software is currently in development, expected to simplify test administration and make it more accessible to researchers and practitioners in various settings. It involves randomized presentation to reduce possible priming effects, a range of manual and default settings and modes (self-paced or timed), tracking of key parameters and video and audio recording throughout the session. Taken together, these features should make the test more objective, reliable and easy to administer.

## 6 Conclusion

The present comparative review provides an overview of the advances in cognitive neuroscience and linguistics that challenge the traditional approach to picture naming as a research and diagnostic method. In particular, the study contributes to the literature by focusing on the theoretical underpinnings of the method, which are rooted in two disciplines: namely, linguistics and cognitive science. While the history of the picture naming paradigm *per se* spans over a hundred years, recent developments in both fields call for a more informed and theoretically grounded interpretation of findings as well as developing new tests.

Our central question was whether the test in the present form is still applicable and what changes it requires to meet not only the modern standards of reliability of findings but also the state-of-the-art understanding of how the speech production works under the experimental conditions of presenting a visual stimulus. In this case PNT could serve as a viable model of language production being a complex cognitive process. “There is nothing more practical than a good theory,” is a statement made by the famous psychologist Kurt Lewin in the 1950s (Kuhn, 1951; Lewin, 1951). A clear theoretical integration not only provides an understanding of how the lexical access is realized through the lenses of cognitive neuroscience and linguistics, but also helps us settle the common starting point for designing better and more informative tests instead of merely pouring new wine into the old sacks of questionable frameworks.

A picture naming test is demonstrated to remain an easy, accessible and efficient method in psycholinguistics. Its current use, however, is limited by two groups of factors. The first, stemming from lack of rigor in test administration, do not concern the properties of the method and can therefore be eliminated with considerable ease: for instance, via an automated, computer-based procedure. Additionally, this would promote the use of picture naming tests in various settings and allow on-the-spot adaptation and tuning if required. The other group includes various issues related to design and interpretation, which were either undetectable or beyond possible improvement at the time most of the popular tests were created. To overcome them, advances in linguistics and neuropsychology should be taken into account; experimental data must be explored in full, and psychometric properties must be assessed thoroughly before stimuli selection. Yet the most significant alterations concern the theoretical foundations of the method itself: namely, the understanding of the language mechanisms in the brain that can manifest themselves in a given speech-based task. To achieve maximum efficiency as a research and clinical method, a picture naming test must be tailored to the needs and characteristics of its target group. In other words, it should be as precise as possible to be able to elicit the minor deficits while avoiding bias. This requires careful consideration of neuroanatomical and neurophysiological correlates of the deficit along with psychosocial, ethnic and linguistic characteristics of the participants. With the improvements suggested above, the picture naming paradigm will remain a valuable diagnostic tool in the XXI century.

## Author contributions

OM: Conceptualization, Investigation, Methodology, Writing – original draft, Writing – review & editing. PM: Conceptualization, Investigation, Methodology, Writing – review & editing. AS: Conceptualization, Investigation, Methodology, Project administration, Writing – review & editing.

## Footnotes

^1^Instead of *grounded cognition*, alternative terms are frequently used in research, in particular *embodied cognition*—the latter term being historically first to arrive in the late XX century (e.g., Varela et al., 1991; Lakoff and Johnson, 1999). While the preference for a particular term is not always accounted for, or both are used within the same work (see Dove, 2022), not all researchers regard them as fully synonymous. For instance, in Barsalou (2020) embodied cognition is restricted to the body proper, while grounding includes social and environmental factors. Since the terminological distinction is less relevant for the purposes of the present article, we choose *grounded cognition* as a term to be used further on.
